# *Pterocarpus santalinus* ameliorates streptozotocin-induced diabetes mellitus via anti-inflammatory pathways and enhancement of insulin function

**DOI:** 10.22038/ijbms.2019.34998.8325

**Published:** 2019-08

**Authors:** Ramy E El-Badawy, Khairy A Ibrahim, Nahla S Hassan, Wael M El-Sayed

**Affiliations:** 1 Medical Lab Specialist, Cleopatra Hospital, Cairo, Egypt; 2 Mammalian Toxicology Department, Central Agriculture Pesticides Lab, Agriculture Research Center, Dokki, Giza, 12618, Egypt; 3 University of Ain Shams, Faculty of Science, Department of Biochemistry, Cairo, Egypt; 4 University of Ain Shams, Faculty of Science, Department of Zoology, Abbassia 11566, Cairo, Egypt

**Keywords:** Fetuin A, IRS-1, JNK, Pterocarpus santalinus, Red sandalwood, SIRT-1

## Abstract

**Objective(s):**

Morbidity and mortality due to diabetes mellitus (DM) result in exorbitant psycho-economical costs, so there is a strong need to create new strategies and drugs for controlling DM. The aim of the current study was to investigate the anti-diabetic effect of the aqueous extract of *Pterocarpus santalinus *on *streptozotocin *(STZ)-induced DM as compared to glustin.

**Materials and Methods:**

Thirty male rats were divided into five groups of six rats each as follows: control; the second group, received the aqueous plant extract (250 mg/kg) orally and daily for three weeks; the third group, was intraperitoneally injected with a single dose of 65 mg/kg of STZ and sacrificed after four weeks; the fourth and fifth groups, were injected with STZ, then after one week these were treated orally with either plant extract or with 3 mg/kg of glustin for three weeks, then sacrificed.

**Results:**

HPLC analysis of the plant aqueous extract showed that it contains many polyphenols and flavonoids. Treatment with STZ resulted in significant reductions in body weight, insulin level, and the expression of Fetuin-A and IRS-1. It also caused significant elevations in glucose, HOMA-IR, glycated hemoglobin, urea, and the expression of JNK and SIRT-1. STZ also caused an extensive β-cell degranulation and decreased cellular density. The aqueous extract of red sandalwood was able to abrogate the deleterious effects caused by STZ and improved the histological architecture of pancreas

**Conclusion:**

The aqueous extract of *P. santalinus* ameliorates diabetes mellitus via anti-inflammatory pathways and enhancement of insulin function.

## Introduction

Diabetes mellitus (DM) is one of the most critical health problems in both developed and developing countries, with about 415 million adult patients estimated worldwide (1). There are three main types of DM; type 1 DM (T1DM), type 2 DM (T2DM), and gestational DM. T2DM is the most common and accounts for about 90% of diabetics. It is characterized by insulin resistance and leads to serious complications in many organs, which affect the quality of life (2).

Diabetes is a multifactorial disease. The liver plays an important role in carbohydrate metabolism. Several hepatokines are involved in glucose homeostasis. Impairment of these hepatic cytokines may lead to impaired insulin sensitivity (3). Fetuin-A is a glycoprotein expressed mainly in the liver (4). It down-regulates the synthesis of pro-inflammatory cytokines and prevents excessive inflammation (5), and may also act as an inflammatory molecule or a positive acute phase reactant (6). Fetuin-A plays a crucial role in the regulation of insulin signaling and risk of T2DM (7). Fetuin-A inhibits the autophosphorylation of tyrosine kinase in insulin receptor substrate 1(IRS-1) by binding to serine of IRS-1, therefore causes a disturbance of normal insulin signaling (8). The silent information regulators (sirtuins) are a family of highly protective proteins. Enzymes encoded by sirtuin (SITR) genes belong to the family of class III histone deacetylases and all of them are NAD^+^-dependent (9). Overexpression of SIRT1 and many SIRT1 activators have beneficial effects on glucose homeostasis and insulin sensitivity in diabetic animal models and humans and down-regulation of SIRT-1 in several cells and tissues have been shown to cause insulin resistance (10). Diabetes mellitus leads to elevation of reactive oxygen species (ROS) in different tissues. Various signal transduction routes such as c-Jun N-terminal kinase (JNK) are stimulated by oxidative stress in different tissues. Stimulation of the JNK pathway, in turn, reduces the insulin gene expression (11). 

Morbidity and mortality due to diabetes result in an exorbitant psycho-economical cost so that there is a strong need to create new strategies and drugs for controlling DM (12). Diet plays an important role in the prevention and management of T2DM (13), and epidemiological and animal studies have shown that consumption of some vegetables can delay or prevent the development of the disease. Phytochemicals, a large group of secondary metabolites of plants used in nutrition, are thought to play a significant role in the health effects of plant-based diets, although the underlying mechanisms of these effects are still unclear (14). 

Medicinal plants act as a natural source for bioactive medicinal compounds. *Pterocarpus santalinus* (red sandalwood) is one of the medicinal plants used in traditional medicine, and is rich in flavonoids and phenols (15). Many previous studies found that different plant extracts have significant antidiabetic effects (16-18). The aim of the current study was to investigate the molecular and inflammatory pathways through which the aqueous extract of *P. santalinus* could exert its anti-diabetic effect on streptozotocin-induced DM in rats. Glustin (pioglitazone) is used to treat T2DM in adults and was used in the current study as a standard drug for comparison. 

## Materials and Methods

***Kits and chemicals***

Streptozotocin (STZ) was purchased from Sigma (St. Louis, MO, USA). Total RNA isolation kit and SYBR Green Master Mix were obtained from Thermo Scientific (USA). Reagents for cDNA synthesis were obtained from Genetix (India). Rat insulin (ELISA) kit was obtained from Elabscience (USA). The kits used for measuring fasting blood glucose (FBG), urea, creatinine, alanine aminotransferase (ALT), aspartate aminotransferase (AST), total cholesterol (T.chol.), triacylglycerols (TAGs), high-density lipoprotein cholesterol (HDL.chol) and glycated hemoglobin (HbA1C) were obtained from BM-Egypt for Medical and Scientific equipment (Cairo, Egypt) and measured as described by the manufacturer.

***Preparation of aqueous extract of Pterocarpus santalinus***

Red sandalwood barks were obtained from a local market (Cairo, Egypt). The plant was identified by a specialized taxonomist. A voucher specimen (CO-1-13) has been deposited in the Department of Botany, Faculty of Science, Ain Shams University, Egypt. The barks were stored in a dry and dark place at room temperature with passive ventilation for 2 weeks. A 100 g of sandalwood bark was powdered using a plant grinder and extracted with 400 ml distilled water for 72 hr at room temperature with continuous shaking. The extract was concentrated under vacuum obtaining a residue of 0.7 g.

***High-performance liquid chromatography (HPLC) analysis of the plant extract***

A high-performance liquid chromatography (HPLC) system equipped with a variable detector 1100 (Aligant, Germany), autosampler, quaternary pump degasser, and column compartment was used to analyze the phenolic and flavonoid contents of the aqueous extract of the plant. The analysis was performed on a C_18 _reverse phase (BDS 5 μm, Labio, Czech Republic) packed stainless-steel column (4 x 250 mm). To determine phenolic acids, 200 mg of the plant water extract was extracted with 10 ml methanol in an ultrasonic bath for 45 min, then the samples were centrifuged for 7 min at 4,200 rpm. The supernatant was filtered through polyamide filter Chromafil AO-45/25. HPLC method started with a linear gradient at a flow rate of 1.0 ml/min with a mobile phase of water/acetic acid (98:2 v/v, solvent A) and methanol/acetonitrile (50:5 v/v, solvent B), starting with 5% B and increasing B to 30% at 25 min, 40% at 35 min, 52% at 40 min,70% at 50 min, and 100% at 55 min. The initial conditions were re-established by 5 min wash with both solvents. All chromatograms were plotted at 280 nm to estimate polyphenols or 330 nm for flavonoids. All components were identified and quantified by comparison of peak areas with external standards.

***Experimental design***

Thirty male rats were divided into five groups of six rats each as follows: the first group was the control, sacrificed after 4 weeks. The second group received the aqueous plant extract (250 mg/kg) orally and daily for three weeks (16). The third group was intraperitoneally (IP) injected with a single dose of 65 mg/kg of STZ (19), and sacrificed after four weeks. The fourth group was injected with a single dose of STZ, then after one week it was treated orally with aqueous plant extract daily for three weeks, then sacrificed. The fifth group was injected with a single dose of STZ, then after one week it was treated orally with 3 mg/kg of a standard drug (glustin) daily for three weeks, then sacrificed. Throughout the experiment, all procedures and experimental protocols were approved by the Institutional Ethics Committee at Faculty of Science, Ain Shams University, and were carried out according to the Guide for the Care and Use of Experimental Animals.

***Real-time PCR analysis***

The levels of gene expression in liver tissue were quantified by quantitative real-time PCR with specific primers for fetuin-A, SIRT-1, JNK, IRS-1, and β-actin as a housekeeping gene. The primers were obtained from Sigma (St. Louis, MO, USA). 

***Primers sequence for the genes***

**Table:** 

Gene	Primers
β -actin	F:TCC TCC TGA GCG CAA GTA CTC T R:GCT CAG TAA CAG TCC GCC TAG AA
Fetuin-A	F:TTGCAGGTTTTGCCCTGCAGA R:GTGGCAGCCAGTCTCTAAGACGT
SIRT-1	F:CTGGGATTGCAGGTATGGACAGCAA R: GCTGAACAAAAGTATATGGACCTATCCG
JNK	F: ATACACCATCGTGTTTGTTTACTACG R: CAGCACGGTTAAAATGGGGAAGAAGT
IRS-1	F:CAG GCA CCA TCT CAA CAA TC R:GTT TCC CAC CCA CCA TAC TG

***Histopathology***

The pancreas was dissected out and fixed in 10% formaldehyde, processed for preparation of paraffin sections and stained with hematoxylin and eosin (H&E) for light microscope examination.

***Statistical analysis***

Significant differences between the mean values were statistically analyzed using one-way analysis of variance (ANOVA) followed by Tukey’s multiple comparison test for the significant interrelation between the various groups. *P*-value was considered significant at *P<*0.05.

## Results

The analysis of the aqueous extract of *P. santalinus* by HPLC showed that the plant contains many polyphenols and flavonoids (Table 1). The major phenols were pyrogallol, E-vanillic, ellagic acid, and catechin, and the major flavonoids were luteo.6-arabinose8-glucose, acacetin, kampferol 3-7-diramoside, naringin, and quercetin.

**Figure 1:**
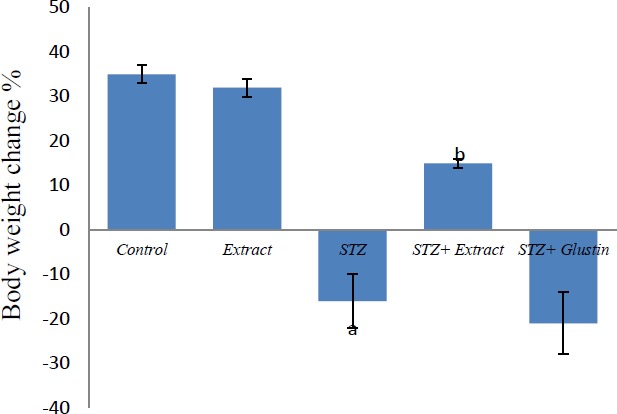
Effect of aqueous extract of *Pterocarpus santalinus* and streptozotocin (STZ) on the body weight change %

Insulin deficiency causes several metabolic alterations. In the current study, the body weight was significantly decreased in STZ group compared to control while the extract alone did not induce any significant change in body weight. The weight of diabetic rats was significantly increased when the animals were treated with the extract compared to diabetic (STZ) group. Glustin did not cause any significant change in the body weight compared to the STZ group (Figure 1).

**Figure 2:**
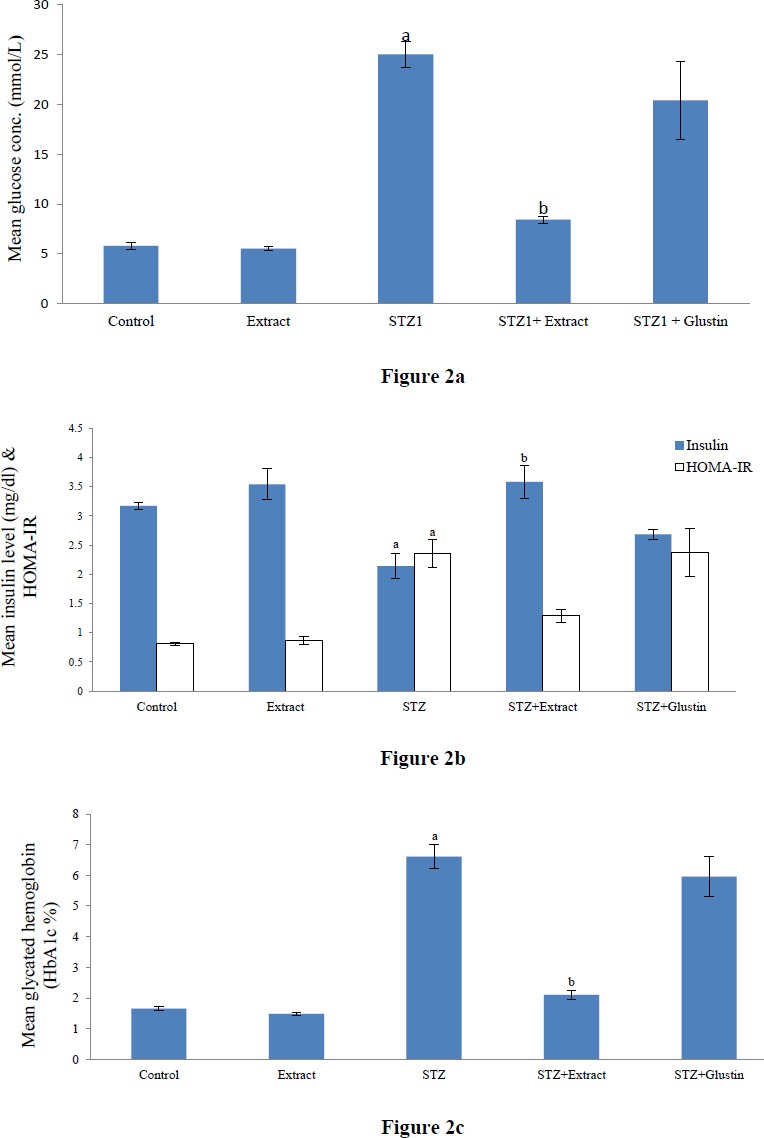
Effect of aqueous extract of *Pterocarpus santalinus* and streptozotocin (STZ) on serum glucose (a); Effect of aqueous extract of *Pterocarpus santalinus* and streptozotocin (STZ) on serum insulin, and homeostatic model assessment (HOMA-IR) (b); Effect of aqueous extract of *Pterocarpus santalinus* and streptozotocin (STZ) on glycated hemoglobin (HbA1c) (c)

In the present study, the STZ-DM was successfully established as indicated by the increase in glucose, HbA1C levels, and HOMA-IR, and the significant reduction in insulin level in the STZ group compared to control (Figures 2 a-c). Treating diabetic rats with the plant extract caused a significant reduction in glucose and HbA1C levels. HOMA-IR was insignificantly decreased and insulin level was significantly elevated compared to the STZ group. On the other hand, glustin failed to ameliorate the previous deteriorations caused by STZ.

The liver and kidney normal functions were assessed. In the current study, the elevation in both ALT and AST activities in serum caused by STZ did not achieve a statistical significance. Treating diabetic rats with the plant extract caused a significant decrease in ALT activity compared to the STZ group, while AST activity was unchanged. Glustin caused a significant decrease in AST activity compared to the STZ group (Table 2). Neither STZ nor the plant extract caused a significant change in creatinine level compared to the control group (Table 2). Streptozotocin caused a significant increase in urea level compared to control. Urea level was significantly decreased in diabetic rats when treated with the plant extract, but glustin failed to reduce the urea level compared to the STZ group (Table 2).

The data showed that neither STZ nor the plant extract caused any significant change in T.chol., TAGs, HDL-chol., LDL-chol., or VLDL levels compared to control. Glustin caused a significant increase in HDL-chol., and significantly reduced the level of LDL-chol. compared to the STZ group (Table 3).

Results of q-PCR showed that streptozotocin caused a down-regulation in the expression of fetuin-A and IRS-1, while there was an up-regulation in the expression of both SIRT-1 and JNK compared to the normal control. Treating the diabetic rats with the plant extract caused a significant down-regulation in the expression of both SIRT1 (65%) and JNK (69%) compared to the STZ group and a significant up-regulation of IRS-1 (~300%) expression level, while fetuin-A expression was insignificantly up-regulated. Glustin caused a significant up-regulation in the expression level of fetuin-A and a significant down-regulation in the expression level of both SIRT1 (30%) and JNK (45%) compared to the STZ group. The plant extract did not cause any significant change in the expression of any genes compared to the control group (Table 4). 

**Figure 3:**
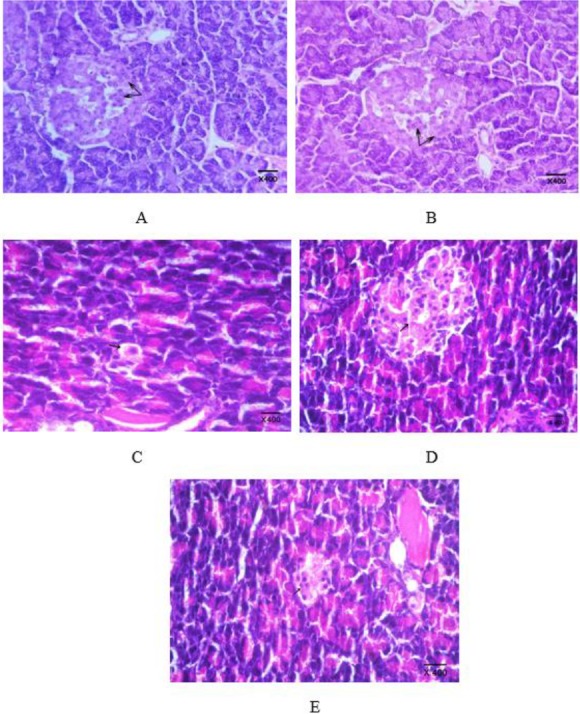
A: A section of pancreas of a rat from normal control group showing no histopathological changes; normal islets of Langerhans and normal pancreatic acini. B: A section of pancreas of a rat treated with the *Pterocarpus santalinus* aqueous plant extract showing no histopathological changes. C: A section of pancreas of a rat treated with streptozotocin showing necrosis and vacuolation of cells of islets of Langerhans and a decrease in the number of the islets. D: A section of pancreas of a rat treated with the plant extract showing no histopathological changes. E: A section of pancreas of a rat treated with the reference drug (glustin) showing mild increase in the number of islets of Langerhans (H&E, X 400)

Histological examination of the pancreas of normal rats or those treated with the plant extract showed no histopathological changes (Figure 3A, B). The pancreas of rats treated with STZ showed degeneration and vacuolation of pancreatic cells and a decrease in the number of β-cells (Figure 1C). Treating diabetic rats with the plant extract caused a marked increase in the number of β-cells (Figure 1D), while glustin caused a mild increase in the number of β-cells (Figure 1E).

## Discussion

The reduction in body weight after STZ-induced DM is attributed to hyperglycemia, hypoinsulinemia, and increased proteolysis (20). The improvement in the body weight by treating diabetic animals with the plant extract may result from its high content of flavonoids. Flavonoids have the ability to stimulate the β-cells to release insulin, ameliorate insulin secretion, decrease hyperglycemia, and restore the body weight (21).

a: indicates a significant difference (*P*<0.05) from control group

b: indicates a significant difference (*P*<0.05) from STZ group

Statistical analysis was performed using one way ANOVA, followed by Tukey-Kramer post-test, n= 6

a: indicates a significant difference (*P*<0.05) from control group

b: indicates a significant difference (*P*<0.05) from STZ group

Statistical analysis was performed using one way ANOVA, followed by Tukey-Kramer post-test, n= 6

**Table 1:** High-performance liquid chromatography (HPLC) analysis of the aqueous extract of Pterocarpus santalinus

Components	Concentration (PPM)
Pyrogallol	2309.8
E-Vanillic	2236.4
Ellagic acid	1963.8
Catechin	177.2
Luteo.6-arabinose8-glucose	1478.1
Acacetin	217.4
Kampferol 3-7-diramoside	82.9
Naringin	44.7
Quercetin	8.6

**Table 2:** Effect of aqueous extract of Pterocarpus santalinus and streptozotocin (STZ) on liver and kidney functions

Groups	ALT (U/L)	AST (U/L)	Creatinine (mg/dl)	Urea (mg/dl)
Control	22.33+1.17	52.33+2.26	0.43+0.01	39.00+2.42
Extract	15.80+1.35	47.17+4.26	0.41+0.02	42.50+3.33
STZ1	31.00+2.16	65.33+5.54	0.45+0.01	73.00+6.50^a^
STZ1+Extract	17.00+1.03^b^	53.67+2.20	0.32+0.01^b^	40.00+2.49^b^
STZ1+Glustin	28.33+2.40	44.00+2.77b	0.41+0.01	77.17+6.67

Statistical analysis was performed using one way ANOVA, followed by Tukey-Kramer post-test, n= 6.

a ^: ^indicates a significant difference (*P<*0.05) from control group.

b ^: ^indicates a significant difference (*P<*0.05) from STZ group.

**Table 3:** Effect of aqueous extract of Pterocarpus santalinus and streptozotocin (STZ) on serum lipid profile

Groups	T.chol. (mg/dl)	TAGs (mg/dl)	HDL-chol. (mg/dl)	LDL-chol. (mg/dl)	VLDL (mg/dl)
Control	73.60+6.39	105.20+19.53	24.40+2.46	28.16+7.57	21.04+3.89
Extract	75.00+6.90	98.00+13.71	27.17+1.77	28.23+6.55	19.60+2.75
STZ	84.00+6.15	96.00+12.47	23.50+2.12	41.30+4.65	19.20+2.49
STZ +Extract	71.17+3.49	86.83+7.51	23.83+1.01	29.98+2.79	17.36+1.50
STZ +Glustin	90.50+4.28	121.33+11.45	50.17+3.16b	16.07+3.63b	24.26+2.29

Statistical analysis was performed using one way ANOVA, followed by Tukey-Kramer post-test, n= 6.

b ^: ^indicates a significant difference (*P<*0.05) from STZ group.

**Table 4:** Effect of aqueous extract of Pterocarpus santalinus and streptozotocin (STZ) on expression of fetuin-A, sirtuin-1 (SIRT-1), c-Jun N-terminal kinase (JNK), and insulin receptor substrate-1 (IRS-1) mRNA

Groups	Fetuin-A	SIRT-1	JNK	IRS-1
(Fold change)				
Extract	0.77+0.04	0.67+0.04	0.89+0.05	1.25+0.19
STZ	0.09+0.01^a^	3.71+0.08^a^	5.60+0.73^a^	0.76+0.08
STZ +Extract	0.15+0.05	1.30+0.14^b^	1.73+0.11^b^	3.01+0.51^b^
STZ +Glustin	1.28+0.37^b^	2.60+0.05^b^	3.06+0.23^b^	1.48+0.10

Statistical analysis was performed using one way ANOVA, followed by Tukey-Kramer post-test, n= 6.

a ^: ^indicates a significant difference (*P<*0.05) from control group.

b ^: ^indicates a significant difference (*P<*0.05) from STZ group.

Streptozotocin has a selective ability to accumulate in pancreatic β-cells by low-affinity glucose transporter 2 (GLUT-2). In addition, STZ causes a generation of reactive oxygen species (ROS) in β-cells. Pancreatic β-cells have a weak antioxidant defense system making them prone to oxidative insult. Therefore, free radicals may cause oxidative damage of nucleic acid (22), apoptosis, and destruction of β-cells (23). This explains why the glucose and HbA1C levels increased and insulin level decreased in STZ-treated animals. HbA1C is considered as a marker to identify the average of plasma glucose concentration, and therefore the degree of glucose control. High level of HbA1C indicates poor control of plasma glucose level (24).

 The ameliorative role of *P. santalinus* aqueous extract against the multiple metabolic disorders caused by STZ could be attributed to the antioxidant activity of polyphenols and flavonoids in the plant. These phytochemicals scavenge free radicals by hydrogen donation or oxygen abstraction and resulting in a more stable state (25). The flavonoids were also shown to regenerate pancreatic β-cells and restore insulin, and to decrease glucose absorption and stimulate insulin secretion (26), and normalize the HbA1C% (17). However, the exact mechanism remains to be elucidated.

The absence of any elevation in hepatic enzyme activity in serum indicates that the doses of the plant extract and other treatments used in the present study were outside the toxic realms. Streptozotocin may cause hepatic injury accompanied by a change in hepatic enzymes (27). It was suggested that the disturbance of ALT and AST caused by STZ is time-dependent (28). In the current study, we have estimated the enzyme activities in the serum at a single time point and did not report any significant change. The specificity of STZ to only destruct pancreatic β-cells without severely affecting hepatic tissue or causing hepatotoxicity was previously reported (28). The plant extract contains many flavonoids and phenols that are known to stabilize the cell membranes and recently, it was shown that luteo.6-arabinose8-glucose (one of the major flavonoids in red sandalwood) stabilized and protected the hepatic cell membranes (29). 

Streptozotocin causes oxidative stress in the diabetic nephrons, thus leads to elevation of urea and creatinine levels, which have been considered as biomarkers of kidney injury (30). Urea level increased in STZ-induced diabetes due to metabolic renal alterations caused by hyperglycemia and hypoinsulinemia such as retarded uptake of amino acids by tissues, a higher rate of proteolysis and a fall in protein synthesis resulting in an elevation of urea production by the liver (19). However, in the current study, the level of creatinine was not changed. Similar results were previously reported (31). Therefore, we believe that the elevated level of urea is due to increased synthesis by the liver rather than impaired secretion by kidneys.

Flavonoids have the ability to reduce the oxidative stress caused by hyperglycemia. Naringin was shown to exert an extra-pancreatic hypoglycemic effect by increasing the utilization of glucose, promoting glycolytic and glycogenic enzymes and decreasing gluconeogenesis (32), therefore resulting in improvement of insulin resistance, decreasing oxidative stress damage and improving kidney functions (22). The low content (~45 ppm) of naringin in the red sandalwood aqueous extract puts some constraints on this explanation. However, a similar action of other flavonoids and phenols cannot be ruled out but this needs further investigation.

Several studies reported that hyperglycemia in STZ-induced diabetic animals caused dyslipidemia (33-35). However, in these studies, the animals were fed a high-fat diet, while in the current study, the animals were fed normal standard pellet diet. In the current study, lipid profile was not affected by the plant extract or by STZ.

Streptozotocin caused a down-regulation in the expression of fetuin-A and IRS-1, while there was an up-regulation in the expression of both SIRT-1 and JNK compared to the normal control. Treating the diabetic rats with the plant extract caused a significant down-regulation in the expression of both SIRT1 and JNK compared to the STZ group and a significant up-regulation of IRS-1 expression level, while fetuin-A expression was insignificantly up-regulated. Glustin caused a significant up-regulation in the expression level of fetuin-A and a significant down-regulation in the expression level of both SIRT1 and JNK compared to the STZ group.

It was previously suggested that fetuin-A is involved in insulin resistance by inhibiting the phosphorylation of tyrosine kinase of the insulin receptor and therefore causes a disturbance of insulin signaling (8). T2DM is considered a low-grade inflammatory disease and causes elevation of the inflammatory cytokines (36). Fetuin-A acts as a negative inflammatory acute phase reactant, and the damage of pancreatic β-cells could lead to down-regulation of fetuin-A as observed in the current study and supported by similar previous findings (37, 38). 

During DM, cells are starved and under these low nutrient conditions, SIRT-1 was up-regulated to raise hepatic glucose production via deacetylation of peroxisome proliferator-activated receptor (PPAR). Reciprocally, overexpression of SIRT1 led to hyperglycemia and impaired glucose tolerance. SIRT1 is involved in a series of critical cellular functions, including circadian rhythms, glucose and lipid metabolism, stress resistance, mitochondrial biogenesis, chromatin silencing, apoptosis, and inflammation (39). Inflammation and glucose homeostasis are important etiologic components of insulin resistance and T2DM. SIRT-1 may also participate in the regulation of insulin secretion and protection of pancreatic β-cells from oxidative stress. SIRT1 can decrease ROS production and apoptosis (40). Therefore, the up-regulation of SIRT1 could be an adaptive mechanism to promote oxidative stress resistance via various mechanisms such as SIRT1-FOXOs pathway (41). 

IRS-1 has many serine and tyrosine binding sites responsible for the action of insulin. Therefore, it has an essential role in normal insulin signaling and response. The metabolic action of insulin in liver tissue occurs in response to the activation of the phosphatidylinositol 3 kinase (PI3K) pathway, in which the insulin receptor is activated by autophosphorylation after binding to insulin (42). The activated insulin receptor subsequently phosphorylates selective tyrosine residues on IRS and induces the binding of IRS-1 and IRS-2 to PI3K, phosphorylating serine and activating protein kinase B (PKB). The activation of PKB is crucial for glucose transport in the liver, muscle and adipose tissue, glycogen synthesis in the liver and muscle tissue, and lipogenesis in the adipose tissue (43). In the present study, the improved insulin sensitivity induced as a result of the plant extract treatment could be mediated by enhanced tyrosine phosphorylation of IRS-1. The aqueous extract of *P. santalinus *can decrease oxidative stress and insulin resistance by suppressing inflammatory related enzymes. Many of the polyphenols and flavonoids found in red sandalwood were previously reported to suppress inflammation and improve insulin resistance (21, 24), which led to a recovery of IRS-1 expression (30). 

Type 2 DM is now considered a chronic inflammatory condition. It is generally accepted that conditions associated with obesity such as increased fatty acids, microhypoxia, endoplasmic reticulum (ER) stress, and elevated cytokines activate pro-inflammatory responses in peripheral insulin target tissues by activating the c-Jun N-terminal kinase (JNK) and inhibiting the nuclear factor κB pathways, which in turn down-regulates insulin signaling (44). Hyperglycemia also causes oxidative stress and enhances the production of ROS from mitochondria. A stress-activated kinase JNK is activated and involved in insulin resistance by binding to IRS-1 in serine, which is an inhibitory site of IRS-1 (45), thus preventing the tyrosine phosphorylation. *P. santalinus *has many flavonoids and polyphenols that exert their anti-diabetic effects via anti-inflammatory and antioxidant pathways. The plant extract was able to suppress the activity of nitric oxide synthase (NOS) resulting in a decrease in nitric oxide (data not shown). Nitric oxide is an important inflammatory mediator.

The biochemical results correlated well with the histopathological study of the pancreas. The pancreas of rats treated with STZ showed degeneration and vacuolation of pancreatic cells and a decrease in the number of β-cells. Treating diabetic rats with the plant extract caused a marked increase in the number of β-cells. STZ causes alkylation and fragmentation of DNA of β-cells and elevated the production of ROS, which has a destructive effect on β-cells due to the weak antioxidant defensive mechanism of these cells (46). The main function of β-cells is to release insulin; this function depends on the mass of β-cells. The mass of cells depends on the equilibrium between the rate of replication and the rate of apoptosis of β-cells. Medicinal plants provide protective and regenerative effects by several mechanisms including increased cellular uptake of glucose, decreased intestinal absorption of glucose, decreased apoptosis and enhancement of pancreatic cell regeneration (47). 

## Conclusion

Streptozotocin was able to induce diabetes mellitus manifested by the reduction in body weight, hyperglycemia, hypoinsulinemia, elevation of glycated Hb and HOMA-IR. STZ was specific in targeting the pancreas causing no hepatotoxicity or nephrotoxicity and did not affect the lipid profile. STZ enhanced the expression of pro-inflammatory and stress markers and reduced fetuin-A expression. It also hindered insulin binding and signal transduction to target cells via reducing the expression of IRS-1. STZ also induced apoptosis and degeneration in β-cells of the pancreas. Taken together, STZ induced insulin resistance, stress and inflammation of pancreas, and disrupted some major hepatokines involved in glucose homeostasis. The aqueous extract of red sandalwood was able to abrogate the deleterious effects caused by STZ and improved the histological architecture of pancreas via anti-inflammatory pathways and enhancement of insulin function. 

## Conflicts of Interest

The authors declare no conflicts of interest.
